# Efficacy of metformin in prevention of paclitaxel-induced peripheral neuropathy in breast cancer patients: a randomized controlled trial

**DOI:** 10.3389/fphar.2023.1181312

**Published:** 2023-07-31

**Authors:** Hala M. Bakry, Noha O. Mansour, Tawfik R. ElKhodary, Moetaza M. Soliman

**Affiliations:** ^1^ Clinical Pharmacy and Pharmacy Practice Department, Faculty of Pharmacy, Mansoura University, Mansoura, Egypt; ^2^ Oncology Center, Medical Oncology Unit, Mansoura University, Mansoura, Egypt

**Keywords:** neuroprotective, nerve growth factor, quality of life, neurotensin, brief pain inventory, FACT-GOG-NTX, chemotherapy, patient reported outcomes

## Abstract

**Background:** Paclitaxel-induced peripheral neuropathy (PN) is a serious clinical problem with no approved drug for prevention. This study aimed to examine the neuroprotective effect of metformin against paclitaxel-induced PN in breast cancer patients.

**Methods:** Patients with confirmed breast cancer diagnosis who were planned to receive paclitaxel were randomized to receive either metformin or placebo. Both groups received the standard chemotherapy protocol for breast cancer. Patients started metformin/placebo 1 week before paclitaxel initiation and continued study interventions thereafter for nine consecutive weeks. The primary outcome was the incidence of development of grade two or more paclitaxel-induced sensory PN. The PN was graded according to the National Cancer Institute Common Terminology Criteria for Adverse Events (NCI-CTCAE). Patients’ quality of life (QoL) was assessed by the Functional Assessment of Cancer Therapy/Gynecologic Oncology Group-Neurotoxicity (FACTGOG-Ntx) subscale. Pain severity was measured by the Brief Pain Inventory Short Form (BPI-SF). Serum levels of nerve growth factor (NGF) and neurotensin (NT) were measured at baseline and at the end paclitaxel treatment.

**Results:** A total of 73 patients (36 in the metformin arm and 37 in the control arm) were evaluated. The cumulative incidence of development of grade two or more PN was significantly lower in the metformin arm (14 (38.9%) than the control arm (28 (75.7%); *p* = 0.001). At the end of paclitaxel treatment, patients’ QoL was significantly better in the metformin arm [median (IQR) FACTGOG-Ntx subscale of (24.0 (20.5–26.5)] compared to the control arm (21.0 (18.0–24.0); *p* = 0.003). The metformin arm showed lower “average” and “worst” pain scores than those detected in the control arm. At the end of the paclitaxel treatment, there was a significant difference in the median serum NGF levels between the two arms, favoring metformin (*p* < 0.05), while NT serum levels were deemed comparable between the two study arms (*p* = 0.09).

**Conclusion:** The use of metformin in breast cancer patients offered a marked protection against paclitaxel-induced PN, which translated to better patient QoL.

**Clinical Trial Registration**: https://classic.clinicaltrials.gov/ct2/show/NCT05351021, identifier NCT05351021.

## 1 Introduction

Paclitaxel is a taxane chemotherapeutic agent that is used in the treatment of breast, ovarian, and lung cancer (Malla et al., 2022). Peripheral neurotoxicity, known as peripheral neuropathy (PN), is a serious clinical problem that is most prominently caused by oxaliplatin (Pachman et al., 2015) and taxanes (Pachman et al., 2016). Taxane-induced PN influences up to 97% of paclitaxel-treated patients and becomes chronic in more than 60% of cases (Tanabe et al., 2013). The acute neurological toxicities related to taxanes occur in a dose-dependent manner, with incidence tending to be higher with cumulative paclitaxel doses of more than 1,000 mg/m^2^ (Rowinsky et al., 1993). The neuropathy is mainly sensory rather than motor or autonomic (Loprinzi et al., 2007). The neuronal degeneration induced by paclitaxel is primarily observed in large myelinated Aβ fibers, leading to impaired sensation in patients. High cumulative doses of paclitaxel cause loss of intraepidermal nerve fibers, resulting in hyperalgesia (Klein and Lehmann, 2021). The initial symptoms of paclitaxel-induced neuropathy, such as numbness, tingling, and/or allodynia, can manifest in the patient’s fingers and toes within 24–72 h post-injection. These symptoms may later progress to affect the patient’s lower leg and wrists in a “glove and stocking” pattern (Dougherty et al., 2004; Argyriou et al., 2008). Symptoms typically begin distally and then continue proximally as the situation worsens (Argyriou et al., 2008; Smith, 2013). A total of 60% of all treated patients manifest chronic paclitaxel-induced peripheral neuropathy. These symptoms cause serious discomfort and might lead to dose reduction, delay, or even termination of treatment in severe cases, limiting therapeutic success (da Costa et al., 2020). Chronic persistence of symptoms severely worsens a patient’s quality of life (QoL). Therefore, the identification of new drugs to prevent neurotoxicity would be a crucial step towards enhancing treatment outcomes in cancer patients. Until now, there have been no FDA-approved drugs for the prevention of chemotherapy-induced PN (Loprinzi et al., 2020). Comprehension of the exact etiology of PN is still lacking, though several potential mechanisms include neuroinflammation, promotion of microtubule polymerization, and oxidative stress (Zajączkowska et al., 2019).

Metformin, a biguanides antidiabetic drug, has an excellent safety profile and well-known pharmacokinetic and pharmacodynamic properties (Nasri and Rafieian-Kopaei, 2014). It has demonstrated beneficial effects in the treatment of various inflammatory diseases (Koh et al., 2014; Cameron et al., 2016; Dehkordi et al., 2019). The discrete neuro anti-inflammatory effects of metformin support its repurposing as a neuroprotective agent in patients with neurodegenerative diseases (Rotermund et al., 2018). Numerous studies have proved that metformin prevents oxidative damage (Esteghamati et al., 2013; Diniz Vilela et al., 2016; Ren et al., 2020). Wang et al. recently reported that metformin also has microtubule-stabilizing and antiapoptotic effects (Wang et al., 2020). Mao-Ying et al. reported that co-administration of metformin with cisplatin or paclitaxel prevented the development of mechanical allodynia and sensory deficits in mice. Specifically, metformin prevented the reduction in density of intra-epidermal nerve fibers, which are associated with a loss of sensory function and increase of pain sensitivity (Mao-Ying et al., 2014). Astrocytes are a type of glial cell that play important roles in maintaining the function of neurons. In response to injury induced by chemotherapy to the peripheral nerves, astrocytes become activated and undergo significant functional changes. The study by Martinez et al. demonstrated that oxaliplatin treatment induced an increase in astrocyte activity and a glial reaction in the spinal cord of mice. However, co-administration of metformin completely prevented this effect, indicating that metformin may have a neuroprotective effect by reducing the glial reaction in the spinal cord (Martinez et al., 2020).

Taken together, metformin might exert a neuroprotective effect against paclitaxel-induced PN. This hypothesis has not been clinically examined. Considering the dearth of approved drugs for preventing chemotherapy-induced PN, this study aimed to assess the protective effect of metformin against paclitaxel-induced PN in patients with breast cancer.

## 2 Patients and methods

### 2.1 Study design and setting

This study was a parallel-group, double-blind randomized controlled trial. Patients were recruited from the Oncology Center at Mansoura University Hospital, Egypt. The study protocol was approved by the Mansoura University Research Ethical Committee (code number: 2021–375). Before patient enrollment, the study protocol was registered at ClinicalTrials.gov (NCT05351021). The study procedures were carried out in agreement with the Declaration of Helsinki. Patients were requested to sign their informed consent before enrollment in the study.

### 2.2 Patients

Patients (aged 18 years and above) with a confirmed diagnosis of breast cancer (Kalli et al., 2018), who planned to receive paclitaxel, and who had an Eastern Cooperative Oncology Group performance (ECOG) status of 0–2 (Oken et al., 1982) were eligible for inclusion in the current study. Patients were excluded if they had pre-existing neuropathy before enrollment, were pregnant/lactating, or had a history of hypersensitivity to metformin. Patients with diabetes, renal impairment (serum creatinine exceeding 1.4 mg/dl/or creatinine clearance less than 45 ml/min), hepatic impairment (aspartate transaminase and alanine transaminase of more than 2.5-fold the upper normal limit), or inadequate bone marrow functions (defined as less than 1,500/mm^3^ absolute neutrophilic count (ANC) or less than 100,000/mm^3^ platelet count) were excluded. Other exclusion criteria were the concomitant use of vitamin supplementation, antidepressants, opioids, and/or systemic analgesics. Patients receiving medications that possibly induce PN, including amiodarone, colchicine, metronidazole, and phenytoin, were also excluded.

### 2.3 Randomization and study interventions

Before randomization, all the recruited patients received the standard anthracycline cyclophosphamide (AC) chemotherapy protocol for breast cancer. The AC regimen comprised doxorubicin (dose/cycle = 60 mg/m^2^) plus cyclophosphamide (dose/cycle = 600 mg/m^2^) for four cycles with 3 weeks in between. The AC protocol was followed by four cycles of dose-dense paclitaxel (dose/cycle = 175 mg/m^2^) with a 2-week period between each two subsequent cycles (Dewidar et al., 2022). One week before paclitaxel treatment initiation, patients were simply randomized in 1:1 ratio either to the intervention group, which received metformin as an adjuvant to paclitaxel, or to the control group, which received placebo. The randomization schedule was concealed in sequentially numbered envelopes. Patients were enrolled by an independent researcher who was not involved in patient care. The group allocation to placebo or metformin was concealed from all the patients and investigators involved in the outcomes assessment. The patients continued the study interventions until the end of the paclitaxel treatment. Before each chemotherapy cycle, the patients received standard supportive treatment regimens of ondansetron plus dexamethasone (8 mg each).

To enhance the patients’ tolerability and minimize side effects, gradual titration of the metformin dose was performed as follows: 850 mg once daily for 1 week, followed by 850 mg twice daily until the end of the treatment. The dose of metformin was selected based on a previous report that indicated the efficacy of a comparable metformin dose (1,500 mg) in the amelioration of neuropathy in patients with colorectal cancer treated with a platinum-based regimen (El-fatatry et al., 2018).

### 2.4 Efficacy outcome

#### 2.4.1 The primary outcome

The primary outcome was comparing the difference in the incidence of development of grade two or more paclitaxel-induced peripheral sensory neuropathy at the end of the paclitaxel treatment between the two arms. The grading of PN was undertaken biweekly, with each cycle of paclitaxel, using the National Cancer Institute Common Terminology Criteria for Adverse Events (NCI-CTCAE) v 5.0 (NCI, 2017). The sensory neuropathy grades range from one to four, where grade 1) is asymptomatic, grade 2) is moderate symptoms, grade 3) is severe symptoms that limit daily self-care activates, and grade 4) is associated with life-threatening consequences.

#### 2.4.2 Secondary outcomes

##### 2.4.2.1 Quality of life (QoL)

QoL was assessed using the Arabic version of the Functional Assessment of Cancer Therapy/Gynecologic Oncology Group-Neurotoxicity (FACTGOG-Ntx) subscale (Calhoun et al., 2003). This tool is a validated and reliable measure for assessment of the impact of PN on a patient’s life (Cheng et al., 2020). It contains 11 questions assessing sensory, motor, and hearing problems. Each item in the Ntx subscale is represented by a Likert scale ranging from zero to four, where zero represents “not at all” and four represents “very much”. Scores are calculated by first reversing the negatively stated items and then summing the resulting item scores. The sum of the individual item scores is multiplied by 11 and then divided by the number of items answered. The Ntx subscale generates total scores ranging from 0 to 44. Higher scores reflect better QoL.

##### 2.4.2.2 Pain severity

Pain severity was evaluated using the Arabic version of the Brief Pain Inventory Short Form (BPI-SF) (Cleeland and Ryan, 1994). Patients were asked to rate their pain on a numerical scale. Each scale was presented as a row of equidistant numbers from zero to ten, where zero indicates “no pain” and ten indicates “pain as bad as you can imagine”. The BPI-SF assesses pain at its “worst”, “least”, “average”, and “now” (current). Clinically, the “average” and “worst pain” are usually recorded to reflect the pain severity.

##### 2.4.2.3 Blood sampling and biochemical analyses

To compare levels of potential markers for neuropathic pain, 5 mL of venous blood was withdrawn at baseline and after the end of the study. The serum was separated by centrifugation, and the supernatant was immediately frozen at −80°C until analysis. Serum concentrations of nerve growth factor (NGF) and neurotensin (NT) were quantified by enzyme-linked immunosorbent assay (ELISA) kits (Human NGF, catalogue number: E-EL-H1205, Elabscience^®^, USA, and Human NT, catalog number: E EL H1886, Elabscience^®^, USA, respectively), as directed by the manufacturers.

### 2.5 Safety outcomes

Study medications were provided to the participants biweekly. They were followed up through direct meetings at every paclitaxel cycle and telephone calls in between cycles to evaluate their adherence and record any adverse effects, particularly those likely related to metformin. Their adherence was evaluated via pill counts. They were considered non-adherent and excluded from the analysis if they administered less than 90% of their study medication.

### 2.6 Patient assessment and follow-up schedule

NCT-CTCAE peripheral neuropathy grading and serum levels of the neuropathy biomarkers were assessed at baseline just prior to paclitaxel initiation and 1 week after the end of paclitaxel therapy (9th week). Quality of life assessment and pain severity evaluation were conducted at the baseline, 6th week, and 9th week.

### 2.7 Sample size calculations

No previous studies were available to estimate the actual effect size of metformin use on the incidence of paclitaxel-induced PN in breast cancer patients. Based on a previous study in patients with colorectal cancer, a large effect size in the primary outcome measure was assumed (El-fatatry et al., 2018). Sample size estimation was performed using G*Power software with a two-sided test at the alpha level of 0.05. It was estimated that a total sample size of 65 patients would have a power of 98% to detect an effect size of 0.5 in the primary outcome (Serdar et al., 2021). To account for possible dropouts, the number of patients in the current study was increased to 76.

### 2.8 Statistical analysis

Statistical analysis was conducted using IBM SPSS^®^ Statistics version 26, IBM corporation software group, USA (IBM^®^ Corp., Armonk, NY, USA). Numerical continuous data were expressed as the mean and standard deviation or median and interquartile range (IQR), as appropriate. Categorical data were expressed as frequency and percentage. Quantitative continuous data were tested for normality using the Shapiro–Wilk test. Every possible comparison between the study groups was performed. For parametric data, the mean values between the two groups were compared using an unpaired Student’s t-test. The Mann–Whitney U test was used to compare non-parametric variables. A comparison of each group over time was carried out using the Freidman test. The chi-square test was used to compare the groups with respect to the categorical data. Survival was analyzed for up to 60 days and was defined as the time (in days) from the first paclitaxel cycle until the development of grade two or more PN or the end of follow-up. Survival analysis was performed using the Kaplan–Meier method, and survival curves were compared using the log-rank test. All *p* values were two sided, and values less than 0.05 were considered significant.

## 3 Results

**FIGURE 1:**
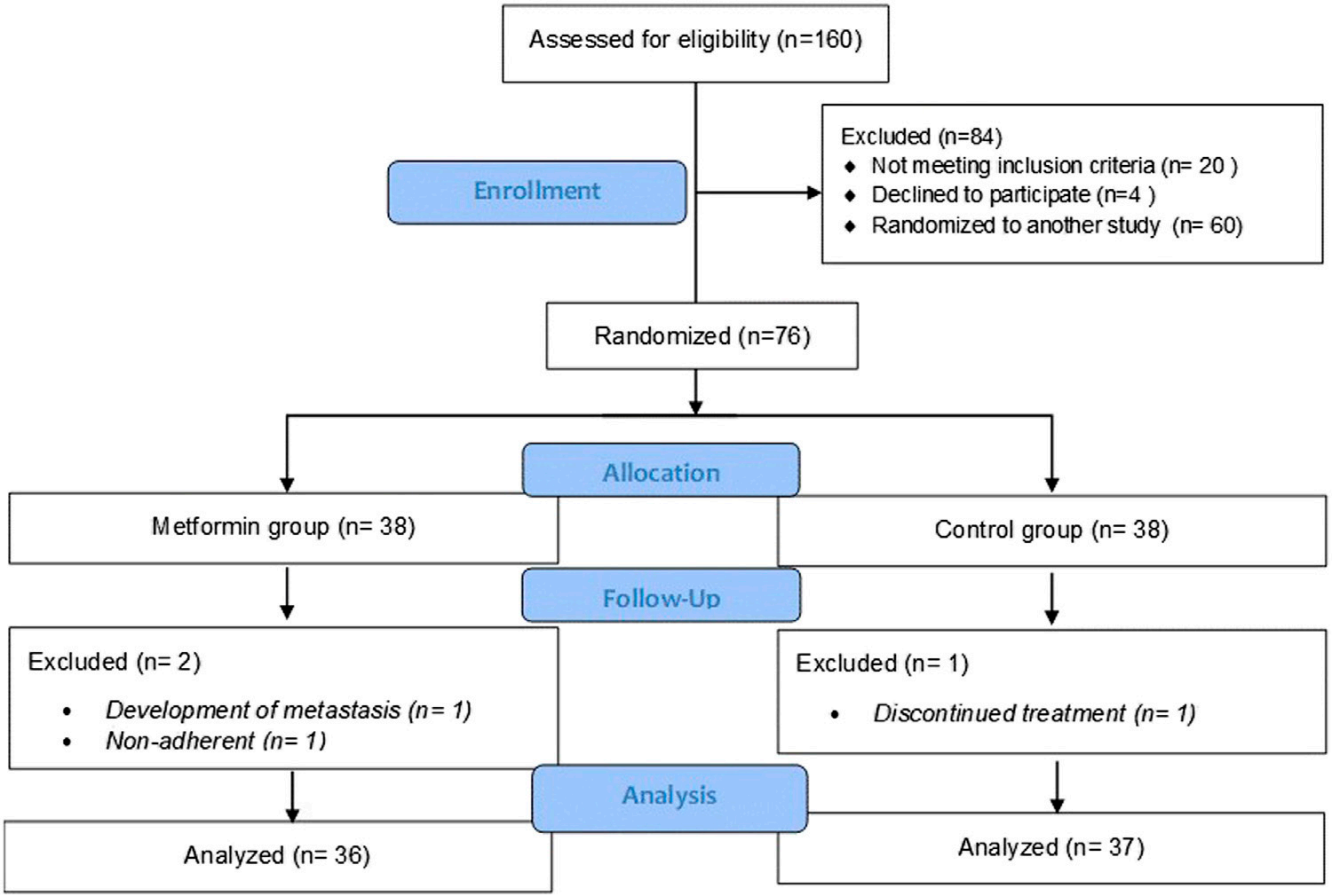
CONSORT flow diagram of patient screening, recruitment, and follow-up.

A total of 160 patients were assessed for eligibility. Of those screened, 76 patients were included in the present study. During the follow-up period, three patients were dropped out due to treatment discontinuation (*n* = 1), non-adherence (*n* = 1), or the development of metastasis (*n* = 1). A total of 73 patients (36 patients in the metformin arm and 37 patients in the control arm) were evaluated for the primary and secondary outcomes of the study. A consort flow diagram of the study is illustrated in Figure 1.

### 3.1 Baseline demographics and biomedical data

The patients’ demographic data were similar for the two studied groups at baseline. Tumor characteristics and laboratory data were also comparable between the two arms of the study (Table 1).

**TABLE 1:** Baseline demographics, clinical, and biomedical data.

Variable	Metformin group *n* = 36	Control group *n* = 37	*p*-value
Age, mean ± SD, years	45.9 ± 8.3	46.6 ± 8.2	0.717 ^a^
BMI, mean ± SD, Kg/m^2^	32.7 ± 6.3	34.5 ± 5.6	0.201 ^a^
BSA, median (IQR), m^2^	1.9 (1.8–2)	1.9 (1.8–2)	0.691^b^
Menopausal state, n (%)			
*Postmenopausal*	11 (30.6%)	16 (43.2%)	0.262^c^
HER 2, n (%)			
* Negative*	18 (50%)	25 (67.6%)	0.127^c^
*Positive*	18 (50%)	12 (32.4%)	
Estrogen receptor			
* Negative*	5 (13.9%)	7 (18.9%)	0.562 ^c^
* Positive*	31 (86.1%)	30 (81.1%)	
Progesterone receptor			
* Negative*	6 (16.7%)	4 (10.8%)	0.467^c^
* Positive*	30 (83.3%)	33 (89.2%)	
Lymph node, n (%)			
* N0*	8 (22.2%)	12 (32.4%)	
* N1*	16 (44.4%)	14 (37.8%)	0.401^c^
* N2*	8 (22.2%)	10 (27%)	
* N3*	4 (11.1%)	1 (2.7%)	
Tumor size, n (%)			
* T0*	1 (2.8%)	0 (0)	
* T1*	10 (27.8%)	6 (16.2%)	0.250^c^
* T2*	19 (52.8%)	24 (64.9%)	
* T3*	4 (11.1%)	7 (18.9%)	
* T4*	2 (5.6%)	0 (0)	
Liver functions; median (IQR)			
Alanine transaminase (IU/L)	19 (14–21.5)	17 (13–20)	0.370^b^
Aspartate aminotransferase (IU/L)	20.5 (15–24)	19 (17–22)	0.580^b^
Serum total bilirubin (IU/L)	0.5 (0.4–0.6)	0.4 (0.3–0.5)	0.046^b^
Serum creatinine; median (IQR), (mg/dL)	0.7 (0.7–0.9)	0.8 (0.7–0.8)	0.699^b^
Blood tests; median (IQR),			
Hemoglobin; (g/dL)	12.8 (12.2–13.5)	12.8 (12.3–13.5)	0.782^b^
White blood cell count; (× 10^9^/L)	7.8 (6.4–9.4)	7.3 (5.6–8.1)	0.075^b^
Platelet count; (× 10^9^/L)	263.5 (212.5–319.5)	237.0 (184.0–264.0)	0.099^b^
Cumulative doses of chemotherapeutic agents; median (IQR), (mg)			
Doxorubicin	456 (420–480)	456 (432–480)	0.691^b^
Cyclophosphamide	4,560 (4,200–4,800)	4,560 (4,320–4,800)	0.691^b^
Paclitaxel	1,330 (1,225–1,400)	1,330 (1,260–1,400)	0.691^b^
Baseline QoL, FACTGOG-Ntx subscale, median (IQR)	41.5 (40.0–42.0)	41.0 (39.0–42.0)	0.202 ^b^
Baseline pain severity	5 (4–6)	6 (5–6)	0.031 ^b^
Baseline Serum levels of NGF, median (IQR)	37.3 (35.7–41.2)	36 (33.6–39.5)	0.054^b^
Baseline Serum levels of NT, median (IQR)	137.9 (110–237.6)	162.1 (120.8–265.4)	0.172^b^

BSA, body surface area; BMI, body mass index; HER2, Human epidermal growth receptor 2; FACTGOG-Ntx, Functional Assessment of Cancer Therapy/Gynecologic Oncology Group-Neurotoxicity; IQR, interquartile range, IU, international unit; NGF, nerve growth factor; NT, neurotensin.

^a^ Independent t-test.

^b^ Mann–Whitney *U* test.

^c^ chi-square test.

### 3.2 Primary outcome

**FIGURE 2:**
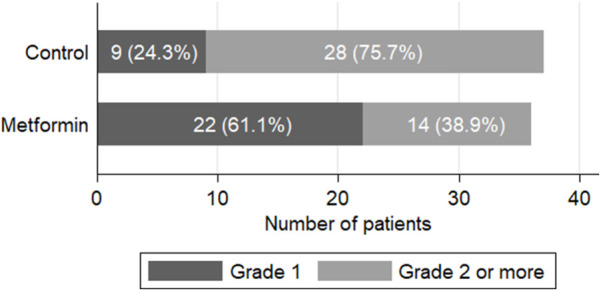
Clinical assessment of paclitaxel-induced peripheral neuropathy at the end of the study (*p* = 0.001).

The cumulative percent of development of grade two or more PN significantly differed between both groups (*p* = 0.001), favoring metformin (Figure 2). Grade two PN was reported in 25 (67.6%) patients in the control arm compared to 13 (36.1%) patients in the metformin arm (*p* = 0.007). Grade three PN was only reported in one patient in the intervention arm *versus* three cases in the control arm with a need for dose delay. None of the patients included in the study developed grade four neuropathy.

### 3.3 Secondary outcomes

#### 3.3.1 Time to develop peripheral neuropathy

**FIGURE 3:**
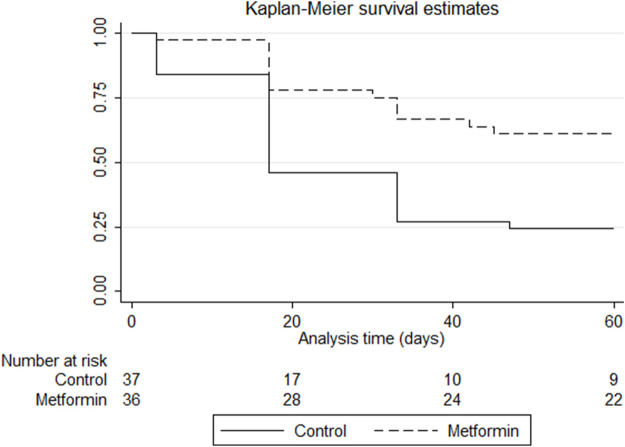
Kaplan–Meier survival estimates of developing grade two or more paclitaxel-induced peripheral neuropathy in the control and metformin arms. *p*-value of log-rank test = 0.0008.

Figure 3 shows the Kaplan–Meier curves for comparison of the time effects. The log-rank test revealed significant difference between the groups (*p* = 0.0008), with patients in the metformin group being less likely to develop grade two or more PN over time than those in the control group. In the control group, 50% of the patients developed grade two or more PN by 17 days, while only 25% of the patients in the metformin group developed this by 30 days.

#### 3.3.2 Quality of life (QoL)

**FIGURE 4:**
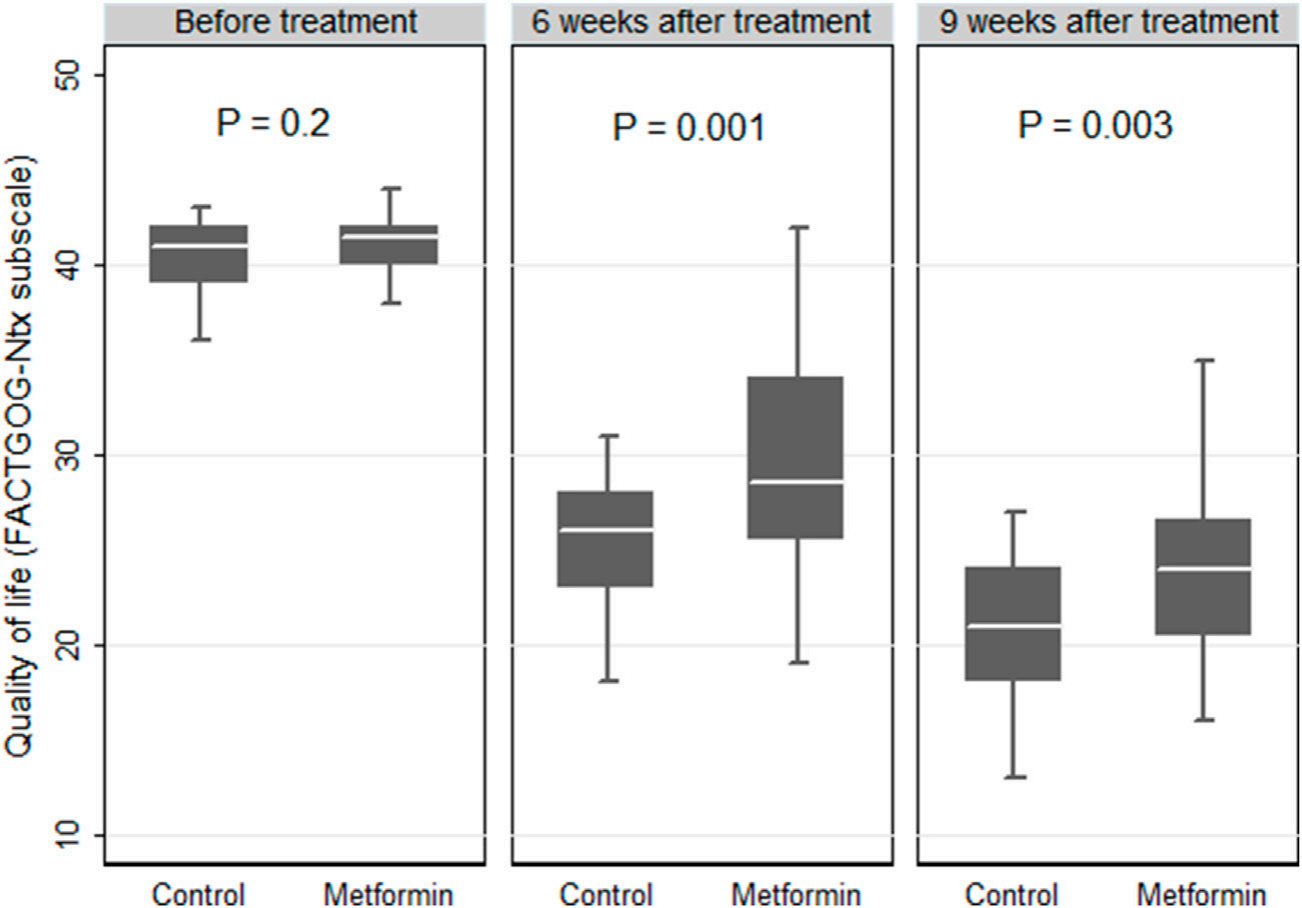
Box plot comparing the quality of life expressed by the Functional Assessment of Cancer Therapy/Gynecologic Oncology Group-Neurotoxicity (FACTGOG- Ntx) in the control and metformin arms.

At baseline, there were no significant differences in the Ntx subscale scores between the two studied arms. In each group, a marked decrease in QoL was observed over time as compared with the baseline (*p* < 0.05 in both groups). At the end of the treatment, comparisons between the two groups revealed statistically higher median (IQR) values of the Ntx subscale scores in the metformin group than those reported in the control group (24 (20.5–26.5) *versus* 21 (18–24), respectively, *p* = 0.003, Figure 4).

#### 3.3.3 Assessment of severity of pain using BPI-SF

**FIGURE 5:**
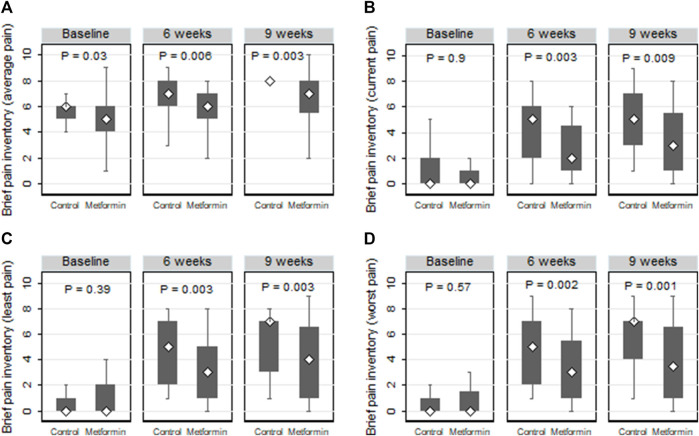
Box plots comparing the average **(A)**, current **(B)**, least **(C)**, and worst **(D)** neuropathic pain according to the brief pain inventory short form (BPI-SF) in the control and metformin arms.

Comparisons of the current, average, least, and worst BPI-SF pain scores across the two arms are represented in Figure 5. At the end of the paclitaxel treatment, the metformin arm showed marked lower median (IQR) “average” and “worst” pain scores than those detected in the control arm (7 (5.5–8.0) *versus* 8 (8–8), *p* = 0.003 and 3.5 (1.0–6.5) *versus* 7 (4.0–7.0), *p* = 0.001, respectively).

#### 3.3.4 Serum biomarkers levels

**FIGURE 6:**
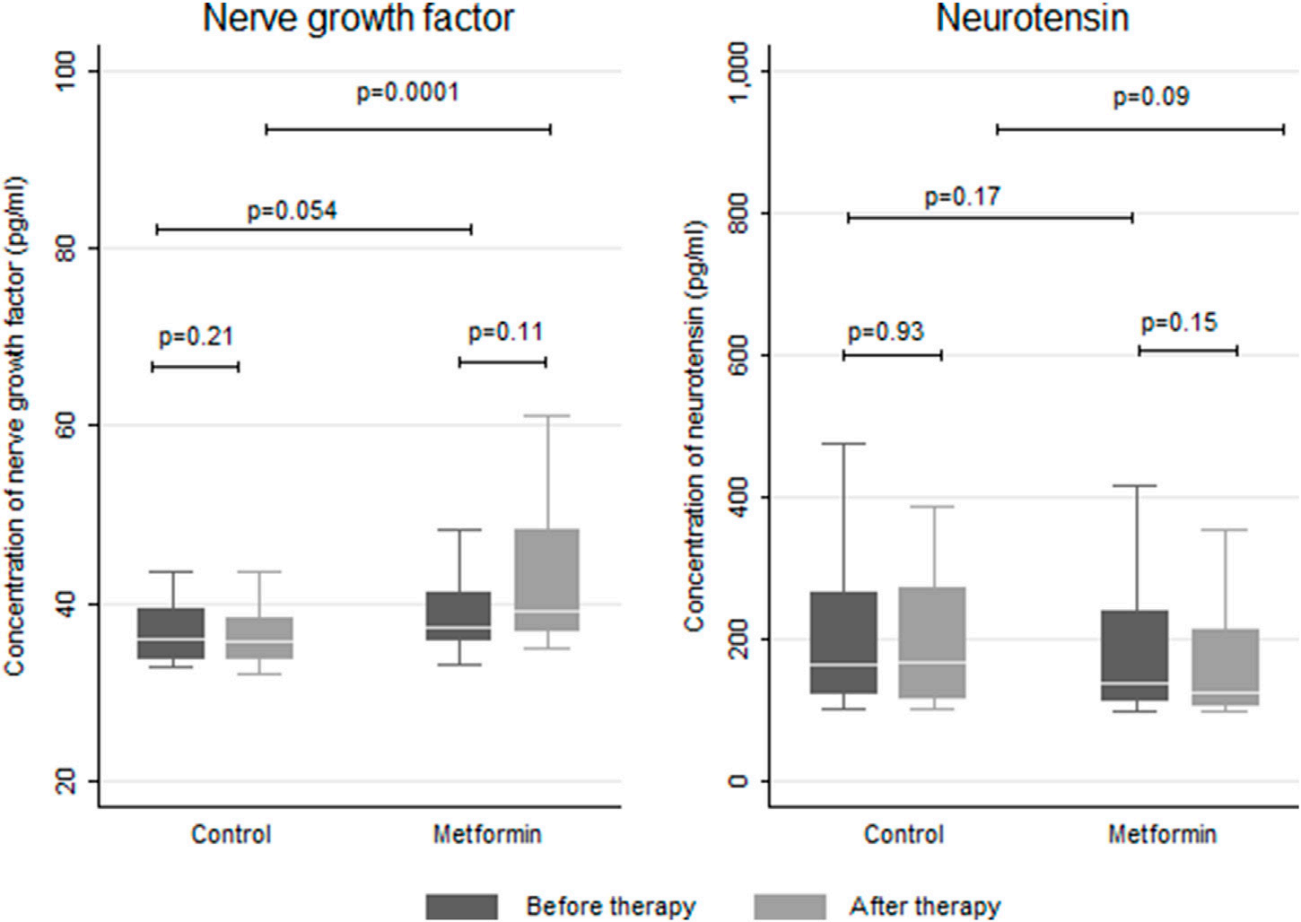
Box plot comparing the biomarker serum levels in the control and metformin arms.

At baseline, the two groups were comparable in terms of NGF and NT serum levels (*p* = 0.054 and 0.172 respectively). At the end of the paclitaxel treatment, there was a significant difference in the median serum NGF between the control and metformin group, favoring metformin (*p* < 0.05). Regarding NT, the serum levels were deemed comparable between the two study arms (*p* = 0.092) at the end of the treatment. The serum levels of NGF and NT are presented in Figure 6.

### 3.4 Safety outcomes

The most frequently experienced adverse effects in both study arms were myalgia, nausea, and headache. As shown in Table 2, the metformin group was comparable to the control group with respect to the frequency of reported side effects, except for diarrhea, which was more frequent in the metformin group (*p* = 0.002). None of the patients in the metformin group discontinued treatment due to this side effect.

**TABLE 2:** Adverse effects reported in the two groups.

Adverse effects, n (%)	Metformin group *n* = 36	Control group *n* = 37	*p*-Value
Headache	15 (41.7%)	19 (51.4%)	0.407^a^
Diarrhea	8 (22.2%)	0 (0)	0.002^a^
Dyspepsia	5 (13.9%)	5 (13.5%)	0.963^a^
Myalgia	23 (63.9%)	30 (81.1%)	0.100^a^
Nausea	21 (58.3%)	21 (56.8%)	0.892^a^
Dizziness	1 (2.8%)	1 (2.7%)	0.984^a^

^a^ Chi-square test.

## 4 Discussion

Prevention of chemotherapy-induced PN is enormously challenging due to varying underlying pathophysiological mechanisms with different chemotherapeutic agents (Desforges et al., 2022). Currently, according to the latest clinical guidelines, there are no effective agents to prevent chemotherapy-induced PN (Loprinzi et al., 2020). The main mechanisms for paclitaxel-induced PN are impairment to the function of the microtubules and breakdown of the transport process in peripheral nerves (Andersen Hammond et al., 2019). The existing solid evidence of the role of metformin in the modulation of these processes stimulates the interest in testing the hypothesis that metformin protects against chemotherapy-induced PN (Mao-Ying et al., 2014; Wang et al., 2020; Zhou et al., 2022).

This is the first randomized study that demonstrates the preventive efficacy of metformin against paclitaxel-induced PN in breast cancer patients. The difference in the incidence of grade ≥2 PN was taken as a primary endpoint. The grading was performed according to the NCI-CTCAE neuropathy grading, the most ubiquitous measure of chemotherapy-induced PN (Li et al., 2022); this approach has been widely used in similar recent clinical trials evaluating other interventions (El-fatatry et al., 2018; Khalefa et al., 2020; Werida et al., 2022; Haroun et al., 2023).

The incidence of NCI-CTCAE grade two or three PN was substantially lower in patients randomized to receive metformin compared to that reported in those allocated to the control group, indicating that metformin had a neuroprotective effect. Moreover, a longer time for the development of PN was shown in the metformin arm than the control arm. Our findings agree with the earlier preclinical evidence where metformin administration protected against paclitaxel-induced mechanical allodynia in a mouse model (Mao-Ying et al., 2014). Clinically, similar findings were reported in colon cancer patients, where metformin use prevented oxaliplatin-induced PN using 1,500 mg metformin daily (60% in the metformin group *versus* 95% in the control group; *p* = 0.009) (El-fatatry et al., 2018).

In search of an underlying mechanism, the protective efficacy reported in the present study is plausibly illustrated by the interference with different pathological contributors. It has been recently demonstrated that activation of adenosine monophosphate-activated protein kinase (AMPK) inhibits necroptosis (Lee et al., 2019), a cell death mechanism associated to several neurodegenerative conditions including paclitaxel-induced PN (Inyang et al., 2019a; Martinez et al., 2020). Metformin, through the activation of AMPK, might promote nerve repair and reduce toxic protein aggregates in sensory neurons (Kong et al., 2020; Demaré et al., 2021). Repurposing metformin as an AMPK activator has been recently shown in randomized trials to improve clinical outcomes in inflammatory diseases (Abdallah et al., 2021) and different neurodegenerative diseases (Asiedu et al., 2016; Markowicz-Piasecka et al., 2017; Demaré et al., 2021). Thus, plausibly, the outcome reported in the present study could be at least partially illustrated by metformin-mediated AMPK activation, which protected against loss of the peripheral nerve endings (Mao-Ying et al., 2014).

The pathogenesis of chemotherapy-induced neuropathies involves changes in the expression of key proteins and signaling pathways. There is consistent evidence suggesting that AMPK activators can induce changes in these pathways and thus may be effective in preventing or treating neuropathies caused by chemotherapy. A study conducted by Pereira investigated the effect of metformin, an AMPK activator, on oxaliplatin-induced sensory peripheral neuropathy. The study found that metformin had a preventive effect on the increase in transcription factors c-Fos and ATF3, which are known to be elevated in response to stress and neuronal injury induced by oxaliplatin in neurons (Pereira et al., 2019). In addition, metformin was found to decrease the activation of microglia and astroglia (Ge et al., 2018; Inyang et al., 2019b), inhibit TRPA1 channels (Wang et al., 2018), and inhibit the mTOR signaling pathway (Melemedjian et al., 2011). These mechanisms are known to play a key role in the pathogenesis of oxaliplatin-related neurotoxicity. Taken together, these findings suggest that metformin targets AMPK to modulate various pathways involved in the pathogenesis of chemotherapy-induced neuropathies, thereby preventing oxaliplatin-associated sensory peripheral neuropathy (Xiao et al., 2012; Carozzi et al., 2015; Di Cesare Mannelli et al., 2015; Duan et al., 2018).

Apart from the AMPK-dependent modulation, our findings might be also illustrated through the putative mitochondrial protective effects of metformin (Du et al., 2022). It acts as a scavenger of reactive oxygen species and subsequently modulates oxidative stress and mitochondrial dysfunction, which are major contributors to neurodegeneration (Areti et al., 2014).

The FACT-Gog-NTx subscale was used for the assessment of the patients’ QoL as a secondary outcome. The 11-item neurotoxicity elements of this questionnaire displayed excellent consistency and validity, with taxane-induced QoL worsening (Cella et al., 2003). A decline in the scores of the FACT-Gog-NTx subscale was observed over time from the baseline to the end of the paclitaxel treatment in both groups. This could be illustrated by the remarkable side effects associated with taxanes such as PN, arthralgia, and myalgia, which significantly worsen a patient’s QoL (Okuyama et al., 2018). However, the FACT-GOC-NTX subscale scores at the end of the treatment were significantly higher in the metformin group than in the control group (24 *vs* 21, *p* = 0.003), indicating better QoL. These findings are in accordance with those of El-fatatry et al., who previously reported higher mean scores of the Ntx-12 questionnaire with metformin use as an adjuvant to a platinum-based regimen in patients with colorectal cancer (24.0 *vs* 19.2, *p* < 0.05) (El-fatatry et al., 2018). On the contrary, the effect of metformin on QoL was evaluated in metastatic breast cancer by Nirula et al. and in early breast cancer by Pimentel et al., and both observed non-significant differences (Niraula et al., 2012; Pimentel et al., 2019). The discrepancy between our results and those studies could be attributed to the use of different non-specific tools in their assessments.

Nerve growth factor is a main neurotrophic factor that supports nerve cell growth and survival (Rocco et al., 2018). This factor is trophic to small-fiber neurons that govern pain and autonomic function. The connection between low NGF serum levels and the occurrence of severe neuropathy has been well recognized in diabetic patients (Anand et al., 1996) and in patients with malignancies as well (Youk et al., 2017). Exogenous NGF administration showed the possibility of preventing paclitaxel-induced PN. At the end of the treatment, a significant increase in the NGF level was evident in the metformin group compared to control group, supporting the potential neuroprotective effect. Our results correlate well with the findings reported by Lós et al., who demonstrated that treatment of diabetic mice with metformin potentiated the NGF expression and attenuated the neuroinflammatory response in the sciatic nerve (Lós et al., 2019).

In terms of metformin tolerability, gastrointestinal adverse effects have been the major concern. In our study, despite metformin being initiated at a low dose and titrated up slowly, a significant increase in the incidence of diarrhea was observed in the metformin arm. However, it did remain tolerable in respect to the self-resolved adverse effects, and none of the patients required treatment discontinuation. Similar findings have been reported with the adjuvant use of metformin in efficacy investigating studies (Barakat et al., 2022; Essa et al., 2022). The incidence of gastrointestinal complications is generally more frequent with metformin immediate-release preparations than with extended-release ones. Hence, it is recommended to use the latter in future studies to improve tolerability. None of the randomized patients in our study experienced hypoglycemia, which is consistent with the established classification of metformin as a drug with negligible risk of inducing hypoglycemia when used as monotherapy (Lipscombe et al., 2018; ElSayed et al., 2023). Nonetheless, caution should be exercised when using metformin in different patient populations who may be more susceptible to hypoglycemia, such as individuals with diabetes who are receiving insulin or sulfonylurea and/or those with renal and hepatic impairment.

Collectively, the previously reported enhancement of taxane antitumor efficacy with adjuvant metformin use and its promising tolerability and affordability, coupled with the findings reported in the present study, present metformin as an ideal preventive agent against paclitaxel-induced PN. However, this study may be limited by the small sample size. Although NCI-CTCAE neuropathy grading represents a valid and robust tool in the grading of neuropathy, it remains limited because of its subjective nature. Therefore, confirmation of our results in future larger trials using objective measures of PN, such as sensory nerve conduction and electromyography, is crucial. The focus on biweekly paclitaxel-based regimens represents another notable limitation. Other taxane-based regimens, such as weekly paclitaxel, docetaxel, or nab-paclitaxel, should be also assessed in future clinical research.

In conclusion, the use of metformin in breast cancer patients was effective in reducing the incidence of paclitaxel-induced PN. The protective effect of metformin was reflected in the patients’ QoL as measured by the FACT-GOG-NTx subscale.

## Data Availability

The raw data supporting the conclusions of this article will be made available by the corresponding author on reasonable request. Requests to access the datasets should be directed to nohamansaur@mans.edu.eg.
